# Timing and risk factors for clinical fractures among postmenopausal women: a 5-year prospective study

**DOI:** 10.1186/1741-7015-4-24

**Published:** 2006-10-09

**Authors:** Antonia CM van Geel, Piet P Geusens, Ivo F Nagtzaam, Cyril MJR Schreurs, Danny JM van der Voort, Paula ELM Rinkens, Arnold DM Kester, Geert-Jan Dinant

**Affiliations:** 1 Care and Public Health Research Institute, Department of General Practice, Maastricht University, PO Box 616, 6200 MD Maastricht, The Netherlands; 2 Department of Methodology and Statistics, Maastricht University, Maastricht, The Netherlands; 3 Department of Internal Medicine and LUC, Diepenbeek, Belgium

## Abstract

**Background:**

Many risk factors for fractures have been documented, including low bone-mineral density (BMD) and a history of fractures. However, little is known about the short-term absolute risk (AR) of fractures and the timing of clinical fractures. Therefore, we assessed the risk and timing of incident clinical fractures, expressed as 5-year AR, in postmenopausal women.

**Methods:**

In total, 10 general practice centres participated in this population-based prospective study. Five years after a baseline assessment, which included clinical risk factor evaluation and BMD measurement, 759 postmenopausal women aged between 50 and 80 years, were re-examined, including undergoing an evaluation of clinical fractures after menopause. Risk factors for incident fractures at baseline that were significant in univariate analyses were included in a multivariate Cox survival regression analysis. The significant determinants were used to construct algorithms.

**Results:**

In the total group, 12.5% (95% confidence interval (CI) 10.1–14.9) of the women experienced a new clinical fracture. A previous clinical fracture after menopause and a low BMD (T-score <-1.0) were retained as significant predictors with significant interaction. Women with a recent previous fracture (during the past 5 years) had an AR of 50.1% (95% CI 42.0–58.1) versus 21.2% (95% CI 20.7–21.6) if the previous fracture had occurred earlier. In women without a fracture history, the AR was 13.8% (95% CI 10.9–16.6) if BMD was low and 7.0% (95% CI 5.5–8.5) if BMD was normal.

**Conclusion:**

In postmenopausal women, clinical fractures cluster in time. One in two women with a recent clinical fracture had a new clinical fracture within 5 years, regardless of BMD. The 5-year AR for a first clinical fracture was much lower and depended on BMD.

## Background

According to the World Health Organization (WHO), osteoporosis is the second leading health problem in the developed world in terms of numbers [1,2], owing to the ageing of the western female population [1-9]. The incidence of fractures is related to the age of the population, and to skeletal and extra-skeletal factors [1-9]. The worldwide lifetime risk of osteoporotic fractures in women is 40%, and because of the ageing of the population, the overall prevalence of osteoporotic fractures is expected to rise considerably [10]. The mortality rate for hip fractures varies between 15 and 30% [10-12]. In 1990, there were 1.7 million hip fractures worldwide, and this could increase to 6.3 million by 2050 [10]. The resulting hospital costs alone amount to over US$3976 million in the European Union, US$500 million in Australia, US$5700 million in the USA, and US$9359 million in Japan [10]. In view of this high morbidity, mortality and economic burden, it is important to identify those groups of patients who are at high risk and for whom interventions to prevent osteoporotic fractures would be most effective [3,4,6-9].

In 1992–1994, a cross-sectional population-based study assessed BMD and other determinants that might influence osteoporosis among 4203 postmenopausal women aged 50 to 80 [7-9]. A random sample of women from the above study was re-examined 5–6 years later, allowing us to study the prognostic value of relevant determinants of experiencing a fracture.

Several researchers have addressed the same subject [13-22], and the WHO is currently developing a fracture risk assessment tool with which family physicians can predict 5-year and 10-year fracture risk [23]. However, data are scarce about the absolute risk (AR) of fractures and the timing of a previous clinical fracture [14-16]. Available studies suggest that fracture risk is highest immediately after a fracture, with the risk of an osteoporotic fracture being 12.0%[14] and that of any fracture being 12.2% during the first 2 years[16]. The risk of morphological vertebral fractures is 19.2% at 1 year after a previous morphological vertebral fracture[15]. The goal of our study was to assess the 5-year AR of incident clinical fractures among postmenopausal women.

## Methods

### Ethics

The ethics review committee of the Maastricht University and the Maastricht University Hospital approved the study (reference number MEC 94-196.1).

### Participants

In total, 20 general practitioners (GPs) from 10 general practice centres participated. The participating general practices were invited based on their familiarity with the rules of 'good clinical practice' and based on their participation in earlier studies. The region can best be described as consisting of two cities surrounded by suburban villages [7].

A random sample of 1686 postmenopausal women, aged between 50 and 80 years, was drawn from the above study population [7-9]. Participating women signed an informed consent form.

### Measurements

The assessments in 1992–1994 started with weight (in kilograms), height (in centimetres), and BMD (measured by a computer-guided DXA instrument; Hologic QDR-1000, Hologic Europe, Brussels, Belgium). Four experienced and specially trained research nurses performed all the measurements. After the measurements, the participants completed a questionnaire, which concentrated on variables possibly related to osteoporosis or low BMD [7-9]. In addition, all women were questioned about their medical history (including fracture history), family history and diet [7].

In the 1998–2000 assessments, one specially trained research nurse, or the principal investigator, completed a questionnaire in the presence of the participant, which recorded the history of fractures. The research assistant confirmed all fractures reported by the patients by searching the medical files in the participating GP centres. In the Netherlands, clinical fractures are treated in hospital and then reported to the patient's GP, who always includes this information in the patient's medical file. In our study, every fracture reported by a patient could thus be confirmed by her medical file.

### Statistical analysis

SPSS software (version 11.0; SPSS Inc., Chicago, IL, USA) was used for the statistical analysis. The dependent variable was the first fracture suffered by a woman after baseline, taking only the first fracture after menopause into account. A power calculation for our main variable (timing of a previous fracture) was performed. This calculation compared two proportions [24].

The hazard of first fracture and the death hazard were used to compute the 5-year probability of fractures. Univariate Cox regression analysis was used to analyse possible predictors of suffering a fracture. Before being entered into the regression analysis, the non-discrete variables were dichotomised at the mean. The cutoff points for coffee intake per day and alcohol intake per week were set at five or more consumptions. The variables 'smoking' and 'sports', both past and present, were dichotomised as yes/no. The resulting significant variables were entered into a multivariate Cox regression to calculate the 5-year probability of fracture.

Further analysis investigated the influence of timing of a previous clinical fracture. A discrete variable was used, categorised as participants with a recent previous fracture (in the past 5 years; n = 68) and participants with an older previous fracture (longer than 5 years previously, but still after menopause; n = 62). The 5-year cutoff point was the optimum compromise between finding a sufficient number of fractures to allow statistical evaluation and minimising the loss to recall [7]. Thereafter, a multivariate subgroup analysis was performed, including the timing of previous fractures. These significant determinants were used to construct algorithms, which included relative risk (RR), AR and 95% confidence intervals (CI).

At baseline (1992–1994), BMD values for lumbar spine were divided into two categories: BMD T-score <-1.0 (including osteoporosis plus osteopenia) and T-score ≥ -1.0, according to WHO criteria [1].

## Results

**Figure 1:**
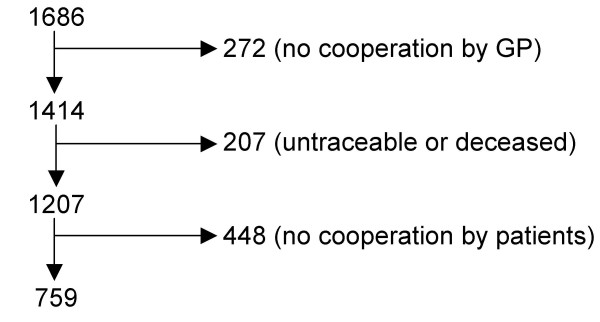
Flow chart of patient inclusion.

Of the 1686 women, 1207 were traceable and alive, and 759 agreed to take part in the follow-up measurement. This corresponded to a response rate of 62.9% of the traceable and surviving women (Figure 1). Non-participating women were compared with participating women in terms of the baseline variables. Age was the only variable that differed between the responders and non-responders; non-participants were on average 3.5 years older than participants.

The power calculation compares a recent previous fracture (n = 68) versus an older previous fracture (n = 62). The two proportions were 0.50 (34/68) and 0.19 (12/62), which resulted in a two-sided power of 0.95. This result showed that the study was sufficiently powered.

The characteristics of the study population at baseline (1992–1994) are shown in Tables 1 (non-discrete variables) and 2 (discrete variables). In total, 95 women (12.5%; 95% CI 10.1–14.9) had a clinical fracture during the 5-year follow-up (Table 3).

The univariate Cox regression showed that low BMD (T < -1.0), increasing age, a history of clinical fracture after menopause, coffee intake, and current or past smoking were significant predictors of a new clinical fracture (Table 4).

As shown in Table 1, there seemed to be some very low values for calcium intake. In an extended univariate Cox regression analysis to investigate the influence of this factor, the lowest quintile and quartile of calcium intake were excluded. This exclusion did not change the results [hazard ratio (HR) = 1.4, 95% CI 0.9–2.2, and HR = 1.3, 95% CI 0.8–2.0, respectively].

These significant determinants were entered into a multivariate Cox regression. The results of this analysis showed that a previous fracture (HR = 5.0, 95% CI 3.4–7.5) was a significant risk factor, and an interaction was found between a previous fracture and BMD. Low BMD contributed significantly only in the presence of interaction, and was therefore an effect modifier. Low BMD was not a significant predictor for patients with a previous fracture (n = 130; HR = 1.5, 95% CI 0.8–2.9), but was for those without (n = 629; HR = 2.9, 95% CI 1.5–5.8).

Table 3 shows the details of 31 hand, foot and other fractures stated. Of these 31 fractures, 12 were caused by a traumatic accident (for example, fall from stairs or bicycle, or fractures incurred during sports). We therefore excluded these 12 fractures from an extended analysis. Multivariate Cox regression again showed that a previous fracture (HR = 5.0, 95% CI 3.2–7.8) was the most significant risk factor. Furthermore, low BMD contributed significantly only in the presence of an interaction, and was a significant predictor only for those without a previous fracture (n = 629; HR = 2.5, 95% CI: 1.2–5.0). Thus, we found that excluding the fractures caused by a trauma did not change the results and they were therefore not excluded from further analysis. 

**Figure 2:**
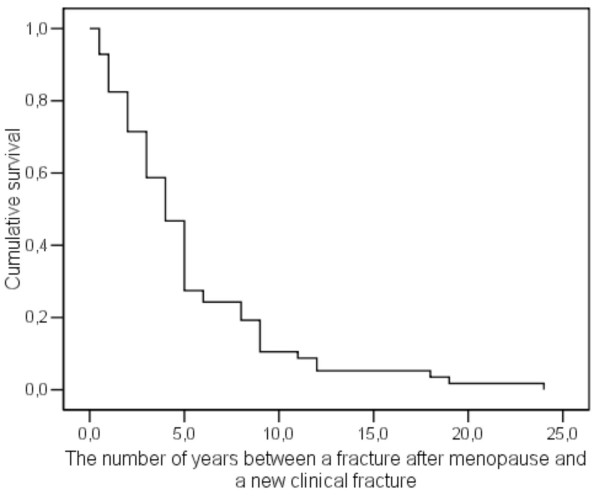
**Cumulative survival plot for postmenopausal women with a previous fracture (n = 130)**. The R^2 ^is 0.98 for an exponential trend line and 0.76 for a linear trend line.

The multivariate subgroup analysis, including the timing of a previous fracture, showed that a recent previous fracture contributed significantly more to the risk of a new clinical fracture than did an older previous fracture (HR = 3.2, 95% CI: 1.7–6.3). Thus, whether a previous fracture was recent was an important risk factor for suffering a new clinical fracture. This result is illustrated in the cumulative survival plot of the postmenopausal women with a previous fracture (Figure 2), and shows clearly that a recent previous fracture was a much more important risk factor than an older previous fracture (R^2 ^= 0.98 for an exponential trend line and 0.76 for a linear trend line).

**Figure 3:**
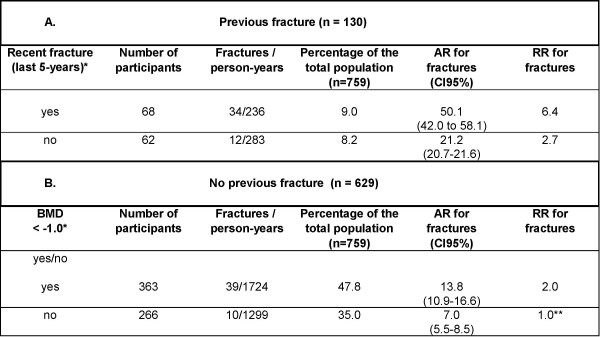
**Algorithm of risk factors for a new clinical fracture**. Algorithm of risk factors for a new clinical fracture, their absolute risks (AR) plus 95% confidence intervals (CI), and relative risks (RR) for the subgroup with (n = 130) and without (n = 629) a fracture history. * Significant risk factors for a new clinical fracture based on multivariate Cox survival analysis; **reference.

The AR for incident clinical fracture was 50.1% (95% CI 42.0–58.1) in women with a recent previous fracture, and 21.2% (95% CI 20.7–21.6) in women with an older previous fracture (Figure 3). In women without a fracture history after menopause, BMD was a significant predictor. The participants without a fracture history and with normal BMD (T ≥ -1.0) had an AR of 7.0% (95% CI 5.5–8.5). This risk was 44.0% lower than that in the total study population (12.5%), and this subgroup represented 35.0% of the total study population. Women without a previous fracture and with low BMD had an AR of 13.8% (95% CI 10.9–16.6), which was 10.4% higher than that of the total study population (Figure 3).

## Discussion

The results show that clinical fractures in postmenopausal women cluster in time. One in two women with a recent previous clinical fracture had a new clinical fracture within 5 years, regardless of BMD. The 5-year AR for a first clinical fracture was much lower and depended on BMD.

The HR of a previous fracture (5.0) is higher than the 2.0 reported in a large meta-analysis[5]; however, our analysis showed that having an older previous fracture (n = 62) led to an HR of 2.5 (95% CI 1.3–4.8), which is closer to the reported AR. The results show that a recent previous fracture is a more important predictor of a new clinical fracture than is an older previous fracture. Owing to effect modification, the algorithm differs between the subgroups with and without a previous fracture, leading to a stronger prediction model per subgroup. This allowed a low-risk group (35.0%) to be identified, consisting of women who can be spared a great deal of unnecessary worry. Nevertheless, 17.2% of the population were at very high risk.

The incidence of vertebral fractures may have been underestimated in our study because only clinical fractures were used. Morphometric fractures that do not come to clinical attention have significance and are not without symptoms[25,26]. Kanis et al[27] showed that the 10-year vertebral probability of morphometric fractures was higher than for clinical fractures alone. Furthermore, morphometric fractures increase the risk of a new clinical fracture significantly and are, therefore, of prognostic significance[15]. BMD, especially measured at the spine, is a strong predictor of a first vertebral fracture[28]. All women in our study with a clinical vertebral fracture (n = 7) had a low BMD. Thus, our AR is generally an underestimate compared with the AR of fractures found when all morphometric fractures were taken into account.

The factors that were independent and significant predictors of fracture risk in our study have been investigated previously, leading to partly comparable results. However, limited data are available about absolute fracture risk and the timing of a previous clinical fracture [14-16] when controlling for most other risk factors. Our study differs from previous in some methodological aspects. Firstly, some studies used prospective data, but presented a risk prediction tool for 1 year[14-16,22,29], which could be too short a time span. Secondly, many studies developed a prediction model specially for hip or vertebral fractures [13,15,17-20], while we included all fractures. Finally, previous results may have differed from ours in terms of geographical factors. For example, the participants of the Dubbo Osteoporosis Study [20] lived in a region with considerably more sun shine than in northern Europe [30,31], which might result in different vitamin D levels.

Age has been documented as a risk factor for fractures in several studies. In our study, age was a significant risk factor for a new clinical fracture only in the univariate analysis. One reason could be that we studied a relatively young population compared with other prospective studies, and the timing of a previous fracture is the main risk for new fractures for this population. All women were aged 50–80 years, with only 12.0% of the population aged 70 years and over, whereas women included in other studies were aged 65 years and over [19-21]. It is possible that our ARs are an underestimate, due to the 3.5-year age difference between the responders and non-responders. However, our results hardly differ from other studies, such as that of Johnell et al[32]. Based on person-years, our absolute fracture risk was 2.7% (95/3542), which is close to the AR reported by Johnell et al (AR: 2.2%; 3694/168 366)[32].

Our study was subject to some limitations. BMD measurement was performed on the lumbar spine, a measurement that can be influenced by osteoarthritis among the elderly. Measurements at the hip could have given additional information, but this had not been performed at baseline (1992–1994). Furthermore, 45 participants (5.9%) received advice about calcium intake and exercise after baseline measurements. Some participants may have heeded this advice, and may therefore have improved their lifestyle. However, the advice differed little from the recommendations regularly made to the general public, on which women may also act. One other factor that may have affected the results is the previous use of medication. However, only the 45 women mentioned above were being treated for osteoporosis (including treatment with a placebo), and excluding these participants from the analysis did not change the results. Finally, our sample size was relatively small in comparison with that in other studies. Johnell et al[32] studied 38 973 men and women (75% women) from 12 prospective cohorts. The total number of osteoporotic and non-osteoporotic fractures in these cohorts was 3694 (AR = 9.5%)[32]. This indicates that even though our sample was relatively small, our absolute fracture risk was still higher than the AR of Johnell's 12 prospective cohorts[32]. Based on person-years instead of numbers of participants, as mentioned previously, our absolute fracture risk was 2.7% (95/3542), which is close to the AR reported by Johnell et al (AR = 2.2%; 3694/168 366)[32].

## Conclusion

The main conclusion of our study is that a recent previous fracture is a relevant factor in detecting postmenopausal women at high risk. One in two women with a recent previous fracture had a new clinical fracture within 5 years, regardless of BMD. This might be interesting information for a variety of professionals: GPs and hospital specialists can use it to quickly and accurately assess which postmenopausal women are at high or low risk, and thus, for which women fracture prevention should be initiated immediately. Future research should try to verify our results using a longer follow-up period.

## Competing interests

The author(s) declare that they have no competing interests.

## Authors' contributions

ACMG is the principal investigator and the main author of the manuscript. PPG and GJD are the supervisors of the principal investigator and responsible for the medical and scientific content of the manuscript. IFN and CMJRS are student investigators and were responsible for a considerable part of the data collection. DJMV is the founder of the cohort and responsible for the preliminary diagnostic studies. PELMR and ADMK are responsible for the statistical analyses of the data. All authors have read and approved the manuscript and agree with publication of their names.

## Pre-publication history

The pre-publication history for this paper can be accessed here:



## Figures and Tables

**Table 1:** Description of the study population at baseline (1992–1994) non-discrete variables (n = 759)

Variable	Mean	SD	Range
BMD (g/cm^2^)	0.94	0.17	0.46–1.59
Age (years)	61.0	6.8	50.1–79.4
Weight (kg)	70.9	11.6	45.0–115.5
Height (cm)	162	6.2	142–183
BMI (kg/m^2^)	27.1	4.2	18–45
Fertility years*	33.0	6.4	5–46
Pregnancies	2.9	2.1	0.0–14
Coffee consumptions/day	5.1	3.3	0.0–30
Alcohol consumptions/week	1.8	5.3	0.0–77
Cigarettes/day smoked in the past	3.4	8.5	0.0–50
Years of smoking in the past	4.8	11.3	0.0–54
Cigarettes/day smoked in the present	3.9	7.7	0.0–50
Years of smoking in the present	9.2	16.3	0.0–57
Calcium intake (mg)	903	407	133–2812

BMD, bone mineral density; BMI, body mass index

*Fertility years = age of last menstruation minus age of first menstruation

**Table 2:** Description of the study population at baseline (1992–1994) discrete variables (n = 759)

Variable	Risk factor present	Percentage
Osteoporosis (T < -2.5)	155	20.4
Osteopenia (-2.5 ≥ T < -1.0))	306	40.3
Normal BMD (T ≥ -1.0)	298	39.3
Fracture history after menopause	130	17.1
Use of birth control pill >5 years	132	17.4
Use of hormones >5 years	48	6.3
Ovariectomy	116	15.3
Hysterectomy	287	37.8
Menopause before the age of 45	255	33.6
Perimenopausal complaints	226	29.8
Family history of osteoporosis	96	12.6
Coffee intake ≥ 5 per day	379	49.9
Alcohol intake ≥ 5 per week	109	14.4
Smoking (past)	157	20.7
Smoking (present)	200	26.4
Sports (present)	469	61.8
Sports (past)	455	59.9
Occupational exercise in the past		
Mild	122	16.1
Moderate	610	80.4
High	24	3.2

BMD = bone mineral density

**Table 3:** Location and number of fractures during the follow-up period

**Location**	**Number**
Vertebra	7
Rib	2
Upper extremities	
Humerus	14
Wrist	30
Hand	5
Other	8
Lower extremities	
Hip	5
Femur	6
Foot	8
Other	10

Total	95

**Table 4:** Univariate Cox regression, hazard ratio and 95% confidence interval (n = 759)

**Variable**	**Hazard ratio**	**95% CI**
BMD ≤ 0.95 g/cm^2^	1.8	1.2–2.8
Osteoporosis*	1.5	0.9–2.3
Osteopenia*	1.3	0.9–2.0
Low BMD*	1.9	1.2–3.1
Age > 60 years	1.7	1.1–2.6
Weight ≤ 70 kg	1.2	0.8–1.8
Height >160 cm	1.0	0.6–1.5
BMI ≤ 27 kg/m^2^	1.1	0.8–1.7
Fertility years ≤ 35 years	1.0	0.7–1.6
Pregnancies > 3	1.4	0.9–2.1
Calcium intake ≤ 900 mg	1.3	0.9–2.0
Fracture history after menopause	5.2	3.4–7.7
Use of birth control pill > 5 years	1.2	0.7–1.9
Use of hormones >5 years	3.3	0.8–13.6
Ovariectomy	1.1	0.7–1.9
Hysterectomy	1.1	0.7–1.7
Menopause below the age of 45	1.2	0.8–1.8
Perimenopausal complaints	1.4	0.9–2.3
Family history of osteoporosis	1.2	0.7–2.1
Coffee intake ≥ 5 per day	1.3	0.9–1.9
Alcohol intake ≥ 5 per week	1.0	0.6–1.8
Smoking (past)†	1.6	1.0–2.4
Smoking (present)†	1.7	1.0–2.8
Sports (past)†	1.0	0.6–1.4
Sports (present)†	1.0	0.6–1.5
Occupational exercise in the past		
Mild	0.3	0.0–2.1
Moderate	0.8	0.5–1.4
High	Reference	Reference

BMD, bone mineral density; BMI, body mass index.

*Osteoporosis = T <-2.5; osteopenia = T >-2.5 and T < -1.0; low BMD = T <-1.0.

†Smoking past and present, and sports past and present were dichotomised as yes/no.
